# Short-Term Outcome of Infants with Biliary Artesia Following Kasai Portoenterostomy

**DOI:** 10.1007/s12098-025-05874-3

**Published:** 2026-01-05

**Authors:** Nourhan Medhat Elhadary, Engy Adel Mogahed, Nevian Nabil, Aya Abdelnaby, Sayed Khedr, Afaf Enayet

**Affiliations:** 1 Department of Pediatrics, Cairo University, Cairo, Egypt; 2 Department of Pediatric Surgery, Cairo University, Cairo, Egypt

**Keywords:** Biliary atresia, Cholestasis, Jaundice, Kasai portoenterostomy, Outcome, Prognosis

## Abstract

**Objectives:**

To assess the short-term outcome of Kasai portoenterostomy (KPE) in infants with biliary atresia (BA) and to identify the prognostic factors for successful KPE.

**Methods:**

The study included 127 infants with BA who presented to the Pediatric Hepatology Unit, Cairo University over a period of 10 y. All patients were followed up for 6-mo post-KPE. Data retrieved from the patients’ files included: history, clinical examination and investigations done at the time of presentation and after 6 mo of KPE. A successful outcome was defined as jaundice clearance after 6 mo of KPE.

**Results:**

Age at KPE ranged from 30–180 d. Marked fibrosis was present more frequently among older patients. Eighty-seven patients (67%) had yellow-colored stools immediately after KPE. Jaundice clearance 6 mo after KPE was achieved in 40 patients (31.5%). Lower age at time of KPE was significantly associated with successful KPE (*P* = 0.03). Steroid therapy post-KPE did not show improvement in jaundice clearance.

**Conclusions:**

KPE had a 6-mo short term successful outcome in one-third of patients with BA. Younger age at KPE is an important prognostic factor to increase the rate of jaundice clearance. Steroids therapy failed to achieve a favorable outcome.

## Introduction

Biliary atresia (BA), the leading cause of neonatal cholestasis, is an obliterative cholangiopathy affecting both intra- and extra-hepatic bile ducts. Kasai portoenterostomy (KPE) attempts in early restoration of bile flow and preservation of the native liver [1]. KPE is the standard initial surgical management of BA and the only warrantor for long-term survival with native liver (SNL). Outcome of KPE and the main prognostic factors influencing it have robustly been evaluated [2]. Liver transplantation (LT) is reserved for those not responding or developing life-threatening complications [3].

Successful KPE with jaundice clearance and long-term (20-y) SNL after KPE remains in the low ranges of 14–44% of patients despite recent advances. So, it is critical to identify and improve the factors affecting the outcome of KPE, aiming to delay and/or reduce the need for LT [4]. This study aimed to assess the success rate and short-term outcomes of KPE in infants with BA, with a secondary objective to identify the prognostic and influential factors for successful KPE.

## Material and Methods

This is a single center retrospective analytical study analyzing the short-term outcome of infants with BA who underwent KPE and were followed-up at the Pediatric Hepatology Unit at Cairo University Pediatric Hospital during the time period from January 2012 to December 2021. This study was evaluated and approved by the Cairo University Research Ethics Committee and was performed in accordance with the Declaration of Helsinki.

The study included all patients with established diagnosis of BA who underwent KPE and followed up for at least 6 mo post-surgery. Demographic data were retrieved from the medical records of the patients as well as histopathological findings of pre-operative liver biopsy and peri-operative data including: Age at time of operation, congenital anomalies detected intra-operative and stool color immediately after operation. Other data retrieved included the patients’ clinical data at the time of presentation, 3 and 6 mo after KPE, abdominal ultrasonographic findings, results of laboratory investigations, as well as the reported complications 6 mo after KPE. Aspartate aminotransferase to platelet ratio index (APRI) score using the formula {Aspartate transaminase (AST)/upper limit of the normal AST range] X 100/Platelet (PLT) count} [5], and Fib-4 score using the formula {[Age (y) X AST (U/L)/PLT (10^9^/L)] X [alanine aminotransferase (ALT) (U/L) (1/2)]} [6] were both calculated at time of presentation.

At authors’ center, steroids are not given prophylactically and were administered as salvage therapy only in patients who re-developed acholic stools following initial bile drainage. The protocol of steroids adopted in authors’ center was administration of oral prednisolone for patients who experienced cessation of bile flow after initial post-operative achievement of bile drainage with colored stools. Oral prednisolone was given at a dose of 1 mg/kg divided in 2 doses after meals. Gradual tapering of steroids dose was started either after achievement of stool coloring or after 1 mo of starting steroids.

Outcome of KPE was assessed by the resolution of jaundice after 6 mo of KPE. Accordingly, patients were divided into 2 groups; the first group with successful KPE who were jaundice free 6-mo post-KPE (total bilirubin < 1.2 mg/dl) and the second group with failed KPE and had persistent jaundice after 6-mo post-KPE (total bilirubin >1.2 mg/dl) [7].

Statistical analysis was performed using the Statistical Package for Social Sciences, version 21 (IBM, Armonk, New York, USA). Quantitative data were described by average and standard deviations (SD) or median and interquartile range (IQR). Qualitative variables were described by frequencies and percentages. Non-normal distributions were compared between two independent groups using the Mann-Whitney U test, while Wilcoson test was used with repeated measurements. Student’s t-test or ANOVA analysis was used to compare averages with a normal distribution. Pearson’s chi-square test was used to compare categorical variables. Analysis of the association between the study variables and the jaundice clearance was performed using the univariate and multivariate logistic regression model. A *P* value of < 0.05 was regarded as significant.

## Results

The total number of patients suspected to have BA who presented to authors’ unit during the study period was 142 cases. Three patients died during the post-operative period with septic shock. Four patients were late presenters (aged 4–5 mo) and had ascites in addition to hepatic dysfunction and were excluded from KPE and were planned for primary LT. For 2 patients with a clinical picture suggestive of BA, the intraoperative cholangiogram revealed patent bile ducts and KPE was not performed. Six patients did not complete the 6-mo follow-up period at authors’ center and their data were not available. In the final analysis, after excluding the aforementioned 15 patients, the present study included 127 patients who fulfilled the inclusion criteria. Sixty-six were males (52%). Their age at the time of presentation to authors’ unit ranged from 7 to 150 d with mean ± SD of 59 ± 21.7 d.

At presentation, all patients had jaundice (100%). Clay stools, as observed by the mother, were present in 85 patients (67%), and as inspected by the physician, in 109 patients (85.8%), while 18 patients (14.2%) had pale yellow stools. Nine patients (7.32%) had their weight and length below 3rd percentile. One hundred and eight patients (85%) had firm hepatomegaly. None of the included patients had ascites at time of presentation. Thirty-five patients (27.6%) had a history of bleeding (from umbilical stump, from puncture site and intracranial hemorrhage). Seven patients had associated congenital cardiac anomalies.

Liver biopsy revealed distorted hepatic architecture in 106 patients (83.5%). Bile duct proliferation was present in all patients and bile plugs were present in 55.1% of cases. Twenty-one patients (16.5%) had a mild degree of hepatic fibrosis, 76 patients (59.84%) had moderate fibrosis and 29 patients (22.8%) had marked fibrosis. Patients with marked fibrosis were older than patients with milder fibrosis degrees, but they did not reach a statistically significant difference.

Age at KPE ranged from 30 to 180 d with mean ± SD, 76.7 ± 23. Thirty-eight patients (30%) had undergone KPE at the age of ≤ 60 d and 89 patients (70%) were aged > 60 d at the time of KPE. Both groups were compared regarding baseline liver functions and liver biopsy findings. ALT, gamma glutamyl transpeptidase (GGT), alkaline phosphatase (ALP) and FIB-4 score were significantly higher in patients who had undergone KPE at the age of > 60 d (Table 1). Eighty-seven patients (67%) had yellow-colored stools immediately after KPE. There was a statistically significant association between the color of stool after operation and after 6-mo of follow-up (*P* = 0.001) denoting that patients who achieved restoration of bile flow immediately after KPE, will probably keep bile flow 6-mo post-operatively. Forty-nine patients (37%) received oral steroids after KPE (in case of cessation of bile flow after an initial restored bile drainage).

**Table 1:** Characteristics of the patients at presentation according to age category at the time of Kasai portoenterostomy (KPE)

Variables	Age category at KPE		*P* value
	≤ 60 d (*n* = 38)	> 60 d (*n* = 89)	
Sex (*n*, %)			
Female	19 (50)	42 (47)	0.8
Male	19 (50)	47 (53)	
Total bilirubin (mg/dl), mean ± SD	11.2 ± 4.5	11 ± 3.4	0.8
Direct bilirubin (mg/dl), mean ± SD	6.5 ± 2.8	6.9 ± 2.7	0.5
AST (IU/L), median (IQR)	152 (195)	171 (121)	0.2
ALT (IU/L), median (IQR)	89 (96)	128 (101)	0.007*
GGT (IU/L), median (IQR)	499 (352)	844 (778)	0.02*
ALP (IU/L), median (IQR)	449 (274)	653 (373)	0.001*
Albumin (g/dl), mean ± SD	3.4 ± 0.5	3.5 ± 0.5	0.7
INR, mean ± SD	1 ± 0.04	1 ± 0.07	0.4
APRI, median (IQR)	0.718 (0.882)	0.836 (0.678)	0.5
FIB-4, median (IQR)	0.005 (0.004)	0.007 (0.005)	0.004*
Degree of fibrosis (in liver biopsy) (*n*, %)			
Mild	8 (20.5)	13 (14.9)	0.2
Moderate	26 (66.7)	50 (57.5)	
Marked	5 (12.8)	24 (27.6)	
Liver architecture (in liver biopsy) (*n*, %)			
Distorted	31 (79.5)	75 (86.2)	0.4
Preserved	8 (20.5)	12 (13.8)	

*ALP* Alkaline phosphatase, *ALT* Alanine aminotransferase, *APRI* Aspartate aminotransferase to platelet ratio index, *AST* Aspartate aminotransferase, *FIB-4* Fibrosis index based on 4 factors, *GGT* Gamma-glutamyl transferase, *INR* International normalized ratio

*P value is statistically significant < 0.05

Clinical examination 6-mo after KPE showed that 13 (13.4%) patients had weight and length below the 3rd percentile. Eighty-seven patients (68.5%) were jaundiced, 2 patients (1.6%) had palmer erythema, 121 patients (95.3%) had hepatomegaly, 108 patients (85%) had splenomegaly, and 27 patients (21.3%) had ascites. Follow-up liver functions of the patients 6-mo post-KPE showed that bilirubin, ALT, AST and GGT significantly improved 6-mo after KPE, while the hepatic synthetic functions decreased but mostly remained within the normal range (Table 2).

**Table 2:** Comparison between laboratory findings of patients with BA at time of presentation and 6 mo after KPE

Variables	At presentation	Six months after KPE	*P* value
Hb (g/dl), mean ± SD	9.8 ± 1.6	9.9 ± 1.6	0.05
Platelet (10^3^/L), median (IQR)	407 (213)	188 (143)	0.001*
TLC (10^3^/L), mean ± SD	12 ± 4	10.9 ± 4	0.01*
T. Bil (mg/dl), median (IQR)	10 (5)	3 (11.3)	0.001*
D. Bil (mg/dl), median (IQR)	6.2 (3.6)	2.2 (8.1)	0.001*
AST (IU/L), median (IQR)	167.5 (133)	125 (122.8)	0.001*
ALT (IU/L), median (IQR)	116 (117.9)	77.7 (87.6)	0.001*
GGT (IU/L), median (IQR)	601 (479)	397 (467)	0.001*
ALP (IU/L), median (IQR)	591 (407)	589 (454)	0.05
Albumin (g/dl) in mean ± SD	3.5 ± 0.5	3.2 ± 0.8	0.001*
INR in mean ± SD	1.03 ± 0.06	1.2 ± 0.35	0.001*

*ALP* Alkaline phosphatase, *ALT* Alanine aminotransferase, *AST* Aspartate aminotransferase, *BA* Biliary atresia, *D. BIL* Direct bilirubin, *GGT* Gamma-glutamyl transferase, *Hb* Hemoglobin, *INR* International normalized ratio, *KPE* Kasai portoenterostomy, *T. BIL* Total bilirubin, *TLC* Total leukocyte count

*P value is statistically significant < 0.05

The range of age of patients with successful Kasai was 35–120 d, while range of age in failed Kasai was 30–180 d. To identify predictors of successful or failed KPE, both groups were compared regarding baseline liver functions, APRI, Fib-4, liver biopsy findings and steroids administration. Univariate analysis revealed that lower age at time of KPE was significantly associated with successful KPE (*P* = 0.03) (Table 3). On the other hand, steroids usage was not associated with improved outcome following KPE. On multivariate logistic regression analysis, only younger age at KPE remained a significant independent predictor of jaundice clearance (*P* = 0.03), while steroid use did not maintain statistical significance (*P* = 0.08).

**Table 3:** Univariate analysis of baseline liver functions and liver biopsy data to predict success of KPE 6-mo post-operatively

Variables	Six-months post-KPE		OR	95% CI	*P* value
	Successful (*n* = 40)	Failed (*n* = 87)			
Sex, *n* (%)					
Female	21 (52.5)	40 (46)	REF	REF	0.49
Male	19 (47.5)	47 (54)	0.77	0.36–1.63	
Age at KPE, days	69.95 ± 17.7	79.75 ± 24.62	0.98	0.96–0.99	0.03*
T. Bil (mg/dl), mean ± SD	10.4 ± 4.5	11.4 ± 3.8	0.93	0.84–1.03	0.19
D. Bil (mg/dl), mean ± SD	6.6 ± 2.8	6.8 ± 2.7	0.72	0.85–1.12	0.07
AST (IU/L), median (IQR)	117 (171)	166 (149)	1.0	1–1.006	0.07
ALT (IU/L), median (IQR)	127 (669)	115 (99)	1.0	0.99–1.004	0.56
GGT (IU/L), median (IQR)	583 (531)	722 (780)	0.99	0.99–1.0	0.07
ALP (IU/L), median (IQR)	626 (428)	586 (401)	1.0	0.99–1.0	0.86
Albumin (g/dl), mean ± SD	3.5 ± 0.6	3.4 ± 0.5	1.55	0.73–3.3	0.25
PT (s), mean ± SD	1 ± 0.04	1 ± 0.07	0.009	0.001–20.3	0.23
APRI, median (IQR)	0.95 (0.79)	0.74 (0.7)	1.45	0.94–2.23	0.97
FIB-4, median (IQR)	0.007 (0.005)	0.006 (0.005)	0.31	0.000–1.43	0.97
Use of steroids, *n* (%)					
No	31 (77.5)	47 (54)	REF	REF	0.01*
Yes	9 (22.5)	40 (46)	0.34	0.14–0.8	
Cholangitis, *n* (%)					
No	25 (62.5)	60 (69)	REF	REF	0.5
Yes	15 (37.5)	27 (31)	1.3	0.61–2.92	
Degree of hepatic fibrosis, *n* (%)					
Mild	10 (25)	12 (13.8)	REF	REF	0.23
Moderate	24 (60)	52 (59.8)	0.59	0.21–1.45	0.06
Severe	6 (15)	23 (26.4)	0.32	0.09–1.07	
Hepatic architecture, *n* (%)					
Distorted	30 (75)	75 (87)	REF	REF	0.09
Preserved	10 (25)	11 (13%)	2.27	0.87–5.9	
Bile plugs, *n* (%)					
No	19 (48.7)	38 (43.7)	REF	REF	0.59
Yes	20 (51.3)	49 (56.3)	0.82	0.38–1.7	

*ALP* Alkaline phosphatase, *ALT* Alanine aminotransferase, *APRI* Aspartate aminotransferase to platelet ratio index, *AST* Aspartate aminotransferase, *CI* Confidence interval, *D. BIL* Direct bilirubin, *FIB-4* Fibrosis index based on 4 factors, *GGT* Gamma-glutamyl transferase, *Hb* Hemoglobin, *INR* International normalized ratio, *KPE* Kasai portoenterostomy, *OR* Odd ratio, *PT* Prothrombin time, *T. BIL* Total bilirubin, *TLC* Total leukocytic count

*P value is statistically significant < 0.05

The most observed complications in present cohort 6-mo after KPE were persistent jaundice (68.5%), hepatomegaly (95.3%), splenomegaly (85%), cholangitis (33.1%), ascites (21.3%), growth failure (10%), itching (9.4%) and bleeding (3.1%).

## Discussion

BA is the most common cause of neonatal cholestasis and end-stage liver disease in the pediatric population. Early intervention with KPE improves patient’s outcome and provides possibility of SNL [8]. The current study aimed to assess the short-term outcome of KPE in infants with BA, and to identify the prognostic factors for successful KPE. One hundred and twenty-seven infants with BA who had undergone KPE over 10 y with at least 6-mo post-operative follow-up were included.

In the present study, prevalence of BA was almost equal between both genders, while other studies described slight predominance of girls [9]. Only 7.32% of the study group had their weight below 3rd percentiles. This contrasts with other reported studies in the United States [10] and Taiwan [11]. The apparently well-nourished appearance of the baby in addition to the misdiagnosis of physiological jaundice attributes to late referral of BA cases to authors’ center. Another cause that may lead to the late referral of these cases, is the inability of caregivers to promptly recognize the difference between normal yellow color of stools and pale or clay color. This emphasizes the importance of introduction of the stool color card to improve the outcome [12].

The routinely administered intramuscular vitamin K injection for every cholestatic patient at the time of initial evaluation in authors’ center might elucidate the low incidence of intracranial hemorrhage in the present cohort (3%). Intracranial hemorrhage is a presentation in significant number of BA patients, and it has variable incidence among countries. Takahashi et al. reported that intracranial hemorrhage occurred in 11.1% of patients with BA [13].

The present results showed that about 77% of index patients had moderate to marked fibrosis and 82.6% had distorted hepatic architecture. The authors did not find any significant association between degree of fibrosis and other parameters. In contrast, Lyu et al. reported that the progressive liver fibrosis group showed lower platelet count, higher APRI score, and higher FIB-4 score compared to the non-progressive liver fibrosis group [14]. The high prevalence of bile plugs (55.1%) in the histopathology may reflect the advanced histological stage at presentation in present cohort, consistent with high rates of moderate to marked fibrosis and late referral patterns in authors’ setting.

Jaundice clearance within 6-mo post-KPE is identified as the only independent predictor of SNL at 2 y [1]. In the present study, jaundice clearance was achieved in 40/127 patients (31.5%). This is comparable to previous studies reported on Egyptian patients as Abdel-Aziz et al. and Yassin et al. who reported 27% and 37% success rates respectively [15, 16]. Meanwhile, there is a wide range of reported jaundice clearance among different countries ranging from 29–82% [10, 17–21].

It is crucial to modulate the factors affecting early outcomes after KPE, aiming to delay or reduce the need for LT [11]. Early age at KPE is the most important prognostic factor for jaundice clearance in patients with BA [22]. In the index study group, younger age was a statistically significant factor associated with successful KPE. In concordance with present findings, several other studies reported that early KPE was associated with favorable outcomes [11, 23–26]. On the other hand, few other studies found that age at KPE did not significantly affect the outcome [16]. In the current study, ALT, GGT, ALP and FIB-4 score were significantly lower in patients who underwent KPE at the age of ≤ 60 d. However, the comparison of baseline laboratory results among different age groups in Hanalioğlu et al. study did not reveal a difference except for the GGT values as lowest GGT levels were reported among patients with age ≤ 60 d [24].

The efficacy of using adjuvant steroids following KPE for BA is still controversial. While many centers tend to use steroids aiming to increase and to keep the bile flow after KPE and to increase the jaundice clearance rate [26–28], other centers did not confirm the recommendation regarding post-operative steroid therapy as they did not affect the long-term outcomes of the jaundice-free survival or improvement in the survival rate with native liver [29, 30]. Similarly, in the present study group, multivariate regression analysis showed that usage of steroids failed to improve jaundice clearance with a statistically significance difference (OR: 0.381, 95% CI: 0.160–0.907; *P* = 0.03). Based upon this finding, the authors do not recommend steroids administration to patients with BA to avoid their unneeded side-effects and they can increase the risk of cholangitis as well as delaying those infants’ vaccination schedule. The present findings should be interpreted in light of the fact that steroids were administered therapeutically, not prophylactically. The controversy regarding prophylactic vs. therapeutic use of steroids after KPE remains, and this variable timing and indication may confound outcomes.

Gad et al. in 2021 reported that early cholangitis was the most reported complication affecting 26.3% of patients [11]. Similarly, during follow-up of index patients for 6-mo after KPE, cholangitis was reported in 33.1% of the cases. However, it was not a significant factor affecting the patients’ outcome.

It is noteworthy that none of the patients in present cohort were referred for LT within the first 6-mo following Kasai portoenterostomy. In Egypt, liver transplantation is performed exclusively through living donor liver transplantation. Given the early age and relatively low body weight of index study population, patients did not yet reach the standard criteria for LT referral during this early post-operative period. Typically, achieving an appropriate weight and stable condition is necessary before considering transplantation to optimize graft-to-recipient size matching and procedural outcomes.

The strength of this study is that it involved documented data of a large number of patients with such a relatively rare disorder, as BA, who underwent KPE by a highly experienced surgical team at a single center. One of the study limitations is its retrospective nature. Moreover, some prognostic factors could not be assessed in the present study, such as type of BA, due to insufficient operative data and unavailability of results of cholangiogram and the size of duct remnants collected from porta hepatis during surgical intervention.

## Conclusions

KPE had 6-mo short-term successful outcome in one-third of patients with BA. Although younger age at KPE is an important prognostic factor to increase the rate of jaundice clearance, KPE should be offered even to late presenting cases as they can have a chance of becoming jaundice free. Steroids therapy failed to achieve a favorable outcome. Predictors can be used objectively to discuss the outcome of KPE and their effect on LT with the parents.
